# Chest Compressions in the Delivery Room

**DOI:** 10.3390/children6010004

**Published:** 2019-01-03

**Authors:** Catalina Garcia-Hidalgo, Georg M. Schmölzer

**Affiliations:** 1 Faculty of Science, University of Alberta, Edmonton, AB T5H 3V9, Canada; garciahi@ualberta.ca; 2 Centre for the Studies of Asphyxia and Resuscitation, Neonatal Research Unit, Royal Alexandra Hospital, Edmonton, AB T5H 3V9, Canada; 3 Department of Pediatrics, University of Alberta, Edmonton, AB T5H 3V9, Canada

**Keywords:** infants, newborn, neonatal resuscitation, chest compressions, delivery room

## Abstract

Annually, an estimated 13–26 million newborns need respiratory support and 2–3 million newborns need extensive resuscitation, defined as chest compression and 100% oxygen with or without epinephrine in the delivery room. Despite such care, there is a high incidence of mortality and neurologic morbidity. The poor prognosis associated with receiving chest compression alone or with medications in the delivery room raises questions as to whether improved cardiopulmonary resuscitation methods specifically tailored to the newborn could improve outcomes. This review discusses the current recommendations, mode of action, different compression to ventilation ratios, continuous chest compression with asynchronous ventilations, chest compression and sustained inflation optimal depth, and oxygen concentration during cardiopulmonary resuscitation.

## 1. Background

Most newborn infants successfully make the transition from fetal to neonatal life without any help [1]. However, an estimated 10–20% of newborns (13–26 million worldwide) need respiratory support [1], which remains the most critical step of neonatal resuscitation [2,3]. Furthermore, 0.1% of term infants and up to 15% of preterm infants (2–3 million worldwide) need extensive resuscitation [4,5,6,7], defined as chest compressions (CCs) and 100% oxygen (O_2_) with or without epinephrine in the delivery room (DR). Despite such care, there is a high incidence of mortality and short-term neurologic morbidity [6,7,8,9,10,11]. The poor prognosis associated with receiving CC alone or with medications in the DR raises questions as to whether improved cardiopulmonary resuscitation (CPR) methods specifically tailored to the newborn could improve outcomes [12,13]. The inability to predict which newborns need CPR and the infrequent use of CPR in the DR have limited the ability of neonatologists to perform rigorous clinical studies aimed at determining the best method for delivering CC to newborn infants [12,13].

## 2. Current Recommendations

The current neonatal resuscitation guidelines recommend to start CCs in a newborn infant if the heart rate remains <60 beats per minute despite adequate ventilation for 60 s [2,3]. CCs should be performed using the two thumb technique and a 3:1 compression to ventilation (C:V) ratio, which consists of 90 chest compressions and 30 inflations [2,3]. This approach is composed of 90 CCs and 30 inflations per minute, with a pause after every third CC to deliver one effective inflation. Inflations and CCs should be synchronized to avoid inadequate oxygen delivery during CCs [2,3]. Furthermore, there is an ongoing debate about how long resuscitation efforts after birth should continue if no heart rate can be detected [2,3]. Only very low quality evidence exists to support of refute this recommendation in infants with a gestational age of ≥36 weeks and an Apgar score of 0 at 10 min. These data suggest that 75/129 infants will die before 22 months of age and 106/129 infants will either die or have moderate/severe neurodevelopmental impairment at 22 months of age [2,3]. Therefore, the current recommendations suggest that it is reasonable to stop resuscitation if a late-preterm or term infant has an Apgar score of 0 at 10 min with an undetectable heart rate. However, given the current evidence, the decision to continue or discontinue resuscitative efforts should be individualized [2,3].

The current recommendation is based on expert opinions and consensus rather than strong scientific evidence [2,3]. Rationales for using 3:1 C:V ratio include (i) a higher physiological heart rate of 120–160 beats/min, and (ii) higher breathing rates of 40–60 breaths/min in newborn infants compared to heart rate and breathing rates adults [12,13]. Ironically, the 3:1 C:V ratio only provides 90 CCs/min, which is considerably less than the normal newborn heart rate of 120–160 beats/min. While the argument may be true that the 3:1 C:V ratio is used in an attempt to match the newborn heart rate and respiratory rate, in actuality, a 3:1 C:V ratio provides considerably lower compressions than the normal newborn heart rate, and, interestingly, is also lower than the recommended 100 compressions per minute in Advanced Cardiovascular Life Support (which is higher than the resting adult heart rate of ~70 beats/min). Furthermore, profound bradycardia or cardiac arrest in newborn infants is usually caused by hypoxia/asphyxia rather than primary cardiac compromise [12,14]; therefore, providing ventilation is more likely to be beneficial in neonatal CCs compared to adult CCs. However, the optimal CC approach to optimize coronary and cerebral perfusion while providing adequate ventilation of an asphyxiated newborn remains unknown [15].

## 3. Mode of Action

During chest compressions, the blood is pumped mechanically through the body until the myocardium becomes sufficiently oxygenated to improve function [16,17,18,19,20,21,22,23]. The generation of blood flow and blood expulsion from the ventricles during CCs is thought to occur by either direct compression of the heart between the sternum and vertebral column (cardiac pump theory) or phasic increases in intrathoracic pressure (thoracic pump theory). The “cardiac pump theory” postulates that CCs directly eject blood from the heart into the circulation with each compression [17]. In comparison, the “thoracic pump theory” states that a phasic increase in intrathoracic pressure produced by compression of the chest creates a pressure gradient between the arterial and venous compartment [21,22,23]. This pressure gradient then serves as the driving force for antegrade blood flow. Optimized CC has been demonstrated to generate 30% of normal organ perfusion, with preferential (>50%) perfusion to the heart and brain [15].

## 4. Using Different Compression to Ventilation Ratios

The current recommendation of using a 3:1 C:V ratio is based on expert opinions and consensus rather than strong scientific evidence [2,3]. Animal studies using cardiac arrest induced by asphyxia in newborn piglets demonstrated that combining CCs with ventilations improves return of spontaneous circulation (ROSC) and neurological outcome at 24 h compared to ventilations or CCs alone [24]. Solevåg et al. performed a study investigating alternating nine CCs and three ventilations in asphyxiated piglets with cardiac arrest, with the hypothesis that nine CCs would generate higher diastolic blood pressure during CCs than only three CCs in a series [25]. However, increasing the number of CCs in a row should not be at the expense of ventilation, hence the ratio of CCs and ventilation was maintained at 3:1. The time to ROSC was similar between the two approaches (150 and 148 s for 3:1 and 9:3, respectively). In addition, there were no differences in diastolic blood pressure during CCs [25]. Similarly, C:V ratios of 3:1 and 15:2 were compared using the same model [26]. Although the 15:2 C:V ratio provided a higher mean CC rate per minute (75 vs. 58 CCs/min for 15:2 and 3:1, respectively), time to ROSC was similar between groups (median time of 195 and 150 s for 15:2 and 3:1, respectively) [26]. Most recently, Pasquin et al. compared 2:1, 3:1, and 4:1 C:V ratios during asphyxia-induced cardiac arrest in newborn piglets and reported no differences in the median (Interquartile range (IQR)) time to ROSC with 127 (82–210), 96 (88–126), and 119 (83–256) s, respectively (*p* = 0.67 between groups), similar oxygen requirements, and similar epinephrine administration between the groups [27]. Furthermore, Traub et al. resuscitated 2–3-week-old piglets after potassium chloride-induced cardiac arrest with C:V ratios of 1:2, 1:3, 1:4, or 1:5 and reported no differences in time to ROSC [28]. Conclusively, these studies suggest that different C:V ratios have similarly ROSC, mortality, and hemodynamic recovery. However, no study has examined short- or long-term neurological outcome, and therefore further studies are needed to address these shortcomings.

## 5. Continuous Chest Compressions with Asynchronous Ventilations

In both adult and pediatric advanced life support, continuous CCs with asynchronous ventilations (CCaV) are recommended after a secure airway has been established [16,17]. In contrast, current neonatal resuscitation guidelines recommend using a coordinated 3:1 C:V ratio if CCs are needed. However, our own observations show that even individuals experienced in resuscitation use CCaV during neonatal resuscitation in the delivery room, most likely due to increased stress [29]. Manikin studies reported higher ventilation rates with 3:1 C:V ratio during simulated CCs compared to higher C:V ratios [30,31,32]. Consequently, the higher ventilation during 3:1 C:V ratio resulted in significantly higher minute ventilation of 191 mL/kg compared to 140 and 77 mL/kg/min with 9:3 and 15:2 C:V ratios, respectively [33]. A further manikin study compared the 3:1 C:V ratio with CCaV using 90 CCs and 30 non-synchronized inflations and reported similar tidal volume (V_T_) measurements of 6.4 and 5.6 mL/kg, respectively. However, minute ventilation was significantly higher in the CCaV group compared to the 3:1 ratio group (221 versus 191 mL/kg/min, respectively) [33]. Schmölzer et al. compared the 3:1 C:V ratio with CCaV in a piglet model of neonatal asphyxia and reported similar median V_T_ (14.7 versus 11.0 mL/kg) and minute ventilation (387 versus 275 mL/kg) [34]. However, there was a trend to reduced median time to ROSC (143 and 114 s for 3:1 ratio and CCaV, respectively) and survival (3/8 and 6/8, respectively) [34]. Most recently, Solevåg et al. compared the 3:1 C:V ratio and CCaV with either 21% or 100% oxygen and reported similar times to ROSC (heart rate ≥100 min^−1^) ranging from 75 to 592 s and mortality of 50–75% between groups [35]. An argument of synchronized CPR is the potential interference of non-synchronized CC with V_T_ delivery, hence impairment of oxygen delivery. However, the study by Schmölzer et al. observed 29% and 25% of manual inflations were similarly affected by CCs during CCaV and 3:1 C:V ratio CCs, respectively [34]. These studies suggest that a similar oxygen delivery can be achieved with coordinated CCs and ventilation at a ratio of 3:1 or CCaV.

Furthermore, the studies by Schmölzer et al. and Solevåg et al. examined hemodynamic changes during CCaV and 3:1 C:V ratio CPR [34,35]. Solevåg et al. reported that immediately after ROSC, piglets resuscitated with CCaV had lower mean arterial blood pressure (at 15 min (*p* = 0.05), but not at 4 h (*p* = 0.20)) [35]. In addition, the myocardial lactate was higher after CPR with CCaV compared to 3:1 C:V ratio CPR, with 1.1 (0.8–1.2) mol/mg protein versus 2.1 (1.3–2.3) mol/mg protein (*p* = 0.05), respectively [35]. In comparison, Schmölzer et al. reported no difference in the percentage changes from normoxic baseline of blood flow index in the pulmonary artery, the common carotid artery, the superior mesenteric artery, and the renal artery [34]. Furthermore, the mean systemic arterial pressure (31 ± 5 and 26 ± 8 mmHg, respectively), mean pulmonary artery pressure (31 ± 7 and 31 ± 9 mmHg, respectively), central venous pressure (23 ± 12 and 19 ± 9 mmHg, respectively), and the mean systemic arterial pressure/mean pulmonary artery pressure ratio (0.98 ± 0.1 and 0.82 ± 0.2, respectively) were similar between 3:1 C:V ratio and CCaV and CPR [34]. These studies suggest that CCaV and 3:1 C:V ratio have different effects on hemodynamics after ROSC.

## 6. Predicting Success of Chest Compressions

During neonatal resuscitation, it is impossible to different between infants who might survive and infants who do not survive [36]. Manley et al. showed videos of the resuscitation of 10 extremely premature infants (mean gestation 26 weeks) to a group of 17 attending neonatologists and 17 neonatal fellows to estimate the likelihood of survival to discharge for each infant at 3 time points: 20 s, 2 min, and 5 min after birth [36]. The observers’ ability to predict survival was poor: 0.61 (95% CI: 0.54–0.67) at 20 s, 0.59 (95% CI: 0.52–0.64) at 2 min, and 0.61 (95% CI: 0.55–0.67) at 5 min [36].

No study has assessed this in newborn infants requiring chest compressions in the delivery room. Furthermore, there are limited data on potential parameters to predict success. Chalak et al. reported that an end-tidal CO_2_ value of 14 mmHg is a predictor for ROSC [37]. Similarly, Chandrasekharan et al. reported that an end-tidal CO_2_ threshold of ≥32 mmHg was 100% sensitive and 97% specific to predicting ROSC in 12 near-term lambs [38]. In addition, Li et al. reported that surviving piglets have significant improved gas exchange and hemodynamics during CPR [39]. Indeed, Li et al. reported that end-tidal CO_2_, the partial pressure of exhaled CO_2_ (PECO_2_), and the volume of expired CO_2_ (VCO_2_) were significantly higher in surviving piglets compared to non-surviving piglets [39]. These studies suggest that end-tidal CO_2_ might be a clinical parameter to guide resuscitation efforts.

Unfortunately, data on hemodynamic parameters are lacking. Schmölzer et al. reported that hemodynamic parameters do not predict success in a post-transitional piglet model [34,35]. Similarly, a neonatal lamb asphyxia model with transitioning circulation reported that hemodynamic parameters do not predict success [40]. However, Vali et al. observed higher lactic acidosis, higher partial arterial O_2_ (PaO_2_), and lower partial arterial CO_2_ (PaCO_2_) in lambs who did not achieve ROSC, which might represent a state of inadequate tissue perfusion and/or mitochondrial dysfunction [40]. Furthermore, Wagner et al. compared hemodynamic parameters using a Millar catheter in surviving and non-surviving piglets [41]. Overall, there were no significant differences in hemodynamic parameters including end-diastolic and developed pressures, dp/dt min, and dp/dt max in surviving and non-surviving piglets [41]. Furthermore, the administration of epinephrine had no effect on heart rate or cardiac output in surviving and non-surviving piglets [41]. These studies suggest that further studies are needed to examine potential hemodynamic parameters that might predict ROSC.

## 7. Optimal Depth

In infants and neonates, current guidelines recommend external compression to a depth of approximately 33% of the anterior-posterior (AP) diameter of the chest, which is relatively greater than that recommended for adults (20% of AP diameter) [2,3]. Studies in adult animals and humans show a positive correlation between receiving adequate CCs and improved outcomes [16]. Adequate CCs are important to achieve adequate cardiac output, however, over-compressing the chest, and therefore leaving inadequate residual chest depth during CC, has its own potential risks. Some of these risks may include rib fractures, cardiac contusion, and other thoracic injuries [12]. Despite the importance of delivering appropriate CCs, adequate CC AP depth has not been rigorously evaluated in neonates.

Manikin studies compared the CC depth using C:V ratios of 3:1, 5:1, and 15:2 during two-minute simulated CPR [30]. Participants had higher and more consistent CC depth during 3:1 C:V, however, the CC rate was lower in CCs with C:V ratio of 3:1 compared to 15:2. In addition, the depth decay during CCs was significantly higher during 5:1 C:V and 15:2 C:V ratios compared to 3:1 C:V ratio [30]. A recent analysis of computed tomography scan images of neonates predicted better ejection fraction (EF) using one-third AP CC depth compared to one-quarter AP CC depth [42]. Furthermore, no subjects receiving the one-third AP CC depth were under-compressed (i.e., had a predicted EF < 50%), whereas 54% were predicted to be under-compressed at the one-quarter AP CC depth. The one-third AP compression depth was much less likely to meet criteria for predicted over-compression than one-half compression depth. This is similar to our own observations in that even individuals experienced at resuscitation do not achieve the required CC depth during neonatal CCs [43,44]. This is concerning, as a decrease in CC depth due to rescuer fatigue can potentially lead to reduced cardiac output and increased mortality.

## 8. Oxygen Concentration During CPR

Oxygen (O_2_) has been used in neonatal resuscitation for over 200 years [45]. Its use spread rapidly in response to reports of brain damage in infants who survived birth asphyxia. The inclusion of skin color in the Apgar score in 1965 further contributed to an increased use of O_2_ in the delivery room [45]. However, over the past decades, 100% O_2_ during delivery room resuscitation has been questioned, as even a brief exposure of asphyxiated infants to 100% O_2_ is associated with adverse effects [46,47,48,49,50,51,52]. Current neonatal resuscitation guidelines recommend 100% oxygen (O_2_) during cardiopulmonary resuscitation (CPR) [2,3], however, the most effective O_2_ concentration during CPR remains controversial.

High concentrations of O_2_ delivery during CPR generate O_2_-free radicals, which play a major role in reperfusion/reoxygenation injury after asphyxia, especially to oxyregulatory tissues (e.g., myocardium). In addition, 100% O_2_ exposure at birth has been associated with increased risk of neonatal mortality and childhood cancer [46,50]. It has been established that depressed infants (with respiratory depression from asphyxia) can be successfully resuscitated with room air, however, its effectiveness in infants with depressed circulation has not been proven. Indeed, several clinical trials and meta-analyses reported that 100% O_2_ during initial mask ventilation at birth resulted in higher rates of (i) time to first breath >3 min (28/284 vs. 60/321 (relative risk (RR) (95% confidence Interval (CI)) 0.53 (0.35–0.8))), (ii) Apgar scores <7 at 5 min (71/288 vs. 102/321 (RR (95% CI) 0.78 (0.6–1.0))), and (iii) death (70/616 vs. 107/659 (RR (95% CI) 0.71 (0.54–0.94))) compared with 21% O_2_ [48,49]. The neonatal resuscitation guidelines have recognized this and recommend air instead of 100% O_2_ during initial mask ventilation [2,3]. However, during CPR, the guidelines continue to recommend an increase to 100% O_2_ [2,3].

Studies in asphyxiated term infants reported that 100% O_2_ during respiratory support in the delivery room resulted in hyperoxemia (PaO_2_, 126 ± 22 mmHg) and increased oxidative stress, but not higher cerebral oxygenation, which does not occur with 21% (PaO_2_, 72 ± 7 mmHg) [52]. Therefore, oxygen delivery during CPR must balance the prevention of tissue damage from O_2_ deprivation with the need to limit the adverse effects of oxidative stress damage from O_2_-free radicals [51,52]. Solevåg et al. resuscitated asphyxiated piglets with 21% vs. 100% O_2_ and reported similar time to ROSC (ranging from 75 to 592 s) with very high mortality rates (50–75%) in both groups [35]. Resuscitation with 21% O_2_ was also associated with improved left ventricular stroke volume after ROSC and lower myocardial oxidative stress (median (IQR) Glutathione disulfide/Glutathione (GSSG/GSH) ratio 0.1 (0.09–0.12) vs. 0.13 (0.11–0.2) *p* = 0.04) compared to 100% O_2_ [35]. In addition, a recent meta-analysis of animal trials (no human studies were identified) reported eight animal studies (*n* = 323 animals) comparing various oxygen concentrations during chest compressions [53]. The pooled analysis showed no difference in mortality rates for animals resuscitated with air vs. 100% oxygen (risk ratio 1.04 (0.35, 3.08), I^2^ = 0%, *p* = 0.94). Time to ROSC was also similar between groups, with a mean difference of −3.8 (−29.7,22) s (I^2^ = 0%, *p* = 0.77). No differences in oxygen damage or adverse events were identified between air vs. 100% oxygen. These results suggest that air has similar time to ROSC and mortality as 100% oxygen during neonatal chest compressions. Randomized trials of air vs. 100% oxygen during neonatal chest compressions are warranted.

## 9. Chest Compressions and Sustained Inflation

An alternative approach of neonatal CCs has been recently described by Schmölzer et al. [54]. The group performed CCs during sustained inflation (SI) (with SI referring to constant high airway pressure while providing CCs) (CC + SI) in asphyxiated piglets and reported significantly improved systemic and regional hemodynamics, tidal volume delivery and minute ventilation, and time to ROSC compared to the 3:1 C:V ratio [54]. CC + SI had an improved recovery, with mean arterial pressure: 51 vs. 31 mmHg; pulmonary arterial pressure: 41 vs. 31 mmHg; mean minute ventilation: 936 vs. 623 mL/kg; and median time to ROSC: 38 vs. 143 s, respectively, compared to the 3:1 C:V ratio [54]. In addition, during CC + SI, a constant lung recruitment and establishment of functional residual capacity was observed (a gain of 2.3mL/kg/CC + SI cycle) [55]. This improvement in V_T_ led to better alveolar oxygen delivery and lung aeration. When using the 3:1 C:V ratio, a cumulated loss of tidal volume of 4.5 mL/kg occurred for each 3:1 C:V cycle [55]. This is concerning, as a loss in V_T_ could cause lung derecruitment, which could hamper oxygenation and therefore ROSC.

Furthermore, the paper by Schmölzer et al. was the first description of passive V_T_ delivery and passive ventilation during neonatal CCs [54,55]. During compression of the chest, volume is exhaled and during chest recoil, gas passively flows back into the lungs. The required distending pressure to achieve passive lung ventilation and adequate tidal volume delivery is ~25 cmH_2_O, which correlated in cadaver piglets (*r* = 0.83, *p* < 0.001), manikins (*r* = 0.98, *p* < 0.001), and when combined (piglets + manikins) (*r* = 0.49, *p* < 0.001) [56]. A similar observation was reported during chest recoil after a downward force was applied to the chest of infants undergoing surgery requiring general anesthesia [57]. Overall, the median (IQR) tidal volume generated was 2.4 (0.8–4.0) mL/kg [57], which suggests that chest recoil generates a distending pressure-dependent tidal volume to allow passive ventilation during CCs. However, in the original study by Schmölzer et al., CCs were performed at a rate of 120/min in the CC + SI group, which is higher than the currently recommended CC rate of 90/min, and this could have contributed to the improved outcomes [54]. Subsequently, we reported that, compared to the 3:1 C:V ratio, CC + SI 90/min resulted in a reduction in median (IQR) time to ROSC (34 (28–156) vs. 210 (72–300) s (*p* = 0.05)), less oxygen (3/8 vs. 8/8 required 100% O_2_, *p* = 0.03), and 3/8 vs. 6/8 piglets received epinephrine (*p* = 0.32) during CCs [58]. Two further randomized piglet trials compared CC rates of 90/min vs. 120/min during CC + SI and CC + SI with either 20 s or 60 s SI and reported similar time to ROSC, survival rates, and respiratory parameters [59,60]. More importantly, during CCs, carotid blood flow, mean arterial pressure, percent change in ejection fraction, and cardiac output were higher in the CC + SI 90/min group compared to CC + SI 120/min [59]. Similarly, Vali et al. reported that CC + SI is feasible in a transitional model of near-term lambs [61]. Most recently, a small pilot trial compared CC + SI and 3:1 C:V ratio CPR in nine preterm infants <32 weeks’ gestation (CC + SI group (*n* = 5; gestational age 24.6 ± 1.3); 3:1 C:V ratio group (*n* = 4; 25.6 ± 2.3) group) [62]. Infants in the CC + SI group had significantly shorter mean ± SD time to ROSC compared to those in the 3:1 C:V ratio group (31 ± 9 vs. 138 ± 72 s, *p* = 0.011) [62]. However, the SI + CC group also had higher use of inotropes (4/5 vs. 0/5), more deaths before discharge (2/5 vs. 0/4), and increased necrotizing enterocolitis (3/5 vs. 1/4) compared to the 3:1 C:V group [62]. The lower diastolic blood pressures reported by Vali et al. [61] in the SI + CC group might have contributed to the higher inotrope use. Furthermore, a recent multicenter trial comparing sustained inflation vs. positive pressure ventilation as initial respiratory support [63] was terminated after 426 infants between 23^+0^–26^+6^ were randomized. The interim analysis identified an increased mortality rate within 48 h after birth with an adjusted relative risk of sustained inflation vs. positive pressure ventilation of 4.73 (95% CI: 1.4, 16.2). While this is concerning, the currently ongoing large multicenter cluster randomized trial comparing CC + SI with 3:1 C:V ratio during neonatal CPR in the delivery room (SURV1VE-trial) is only recruiting infant >28 weeks’ gestation in order to reduce potential harm to this vulnerable population (https://clinicaltrials.gov/ct2/show/NCT02858583).

## 10. Use of Animal Models

Animal models are used to understand the science of normal and pathophysiological states in humans and have given rise to much of our current understanding of normal human biology and the mechanisms of disease [13,64,65]. To examine neonatal CPR, studies have used piglets and sheep models [13,64,65]. Piglets (domestic or miniature) are the most commonly used animal models for neonatal CC studies. Advantages of the piglet model include (i) distribution of coronary blood, (ii) blood supply to the conduction system, (iii) histologic appearance of the myocardium, and (iv) biochemical and metabolic responses to ischemic injury similar to human pathophysiology [13,64,65,66]. Disadvantages of the piglet model include (i) large inter-individual variation, making detection of significant differences caused by interventions challenging, and (ii) large litter size, which makes the development of transitional models challenging [13,64,65]. Sheep have been extensively used to study physiological changes and resuscitation techniques during perinatal transition [67,68,69,70,71]. Advantages of newborn sheep include (i) usefulness in examining lung mechanics during perinatal transition, (ii) large body mass and small litter size, and (iii) similar physiology and anatomy to human newborn infants [13,64,65]. Disadvantages of the sheep model include (i) large inter-individual variation, (ii) precocious maturation as compared to human infants of 44 weeks post-conceptual age, and (iii) V-shaped chest, which potentially affects the delivery of antero-posterior CCs compared to a human newborn [13,64,65].

## 11. Future Directions

The current evidence for chest compressions in newborn infants is primarily based on animal data and expert opinions. There is a need for basic/translational evidence before high quality clinical trials can be performed. Before changes are made to guidelines, more studies are needed that show how CC protocols vary among and effect rescuers. Additionally, studies must include long-term neurodevelopmental outcomes in order to determine the possible long-term effects of neonatal CPR.

## 12. Conclusions

Successful cardiopulmonary resuscitation from cardiac arrest requires the delivery of high-quality chest compressions encompassing several factors including optimal compression:ventilation ratio, depth of chest compression, and oxygen concentration during cardiopulmonary resuscitation. Clinical trials in this area are urgently needed.

## Abbreviations/Nomenclature

C:V ratio	Compression to ventilation ratio
CC	Chest compression
CCaV	Continuous chest compressions with asynchronous ventilations
CPP	Coronary perfusion pressure
CPR	Cardiopulmonary resuscitation
DR	Delivery room
PPV	Positive pressure ventilation
ROSC	Return of spontaneous circulation
SI	Sustained inflation
